# Stepping into the Void: Lessons Learned from Civil Society Organizations during COVID-19 in Rio de Janeiro

**DOI:** 10.3390/ijerph20085507

**Published:** 2023-04-14

**Authors:** Rodrigo Curty Pereira, Susan J. Elliott, Pablo Llaguno Cárdenas

**Affiliations:** 1 Geography and Environmental Management, University of Waterloo, Waterloo, ON N2L 3G1, Canada; 2 International Relations and Political Science, Tecnológico de Monterrey, Monterrey 64849, NL, Mexico

**Keywords:** COVID-19, civil society organizations, water, WASH, hygiene

## Abstract

Brazil experienced some of the highest rates of COVID-19 globally. This was complicated by the fact that 35 million of its citizens have limited access to water, a primary resource necessary to stem the spread of infectious diseases. In many cases, civil society organizations (CSOs) stepped into this void left by responsible authorities. This paper explores how CSOs in Rio de Janeiro helped populations struggling with access to water, sanitation, and hygiene (WASH) during the pandemic, and what coping strategies are transferable to similar contexts. In-depth interviews (*n* = 15) were conducted with CSO representatives in the metropolitan region of Rio de Janeiro. Thematic analysis of the interviews revealed that COVID-19 exacerbated pre-existing social inequities among vulnerable populations, undermining their ability to protect their health. CSOs provided emergency relief aid but faced the counterproductive actions of public authorities who promoted a narrative that diminished the risks of COVID-19 and the importance of non-pharmacological interventions. CSOs fought this narrative by promoting sensitization among vulnerable populations and partnering with other stakeholders in networks of solidarity, playing a vital role in the distribution of health-promoting services. These strategies are transferrable to other contexts where state narratives oppose public health understandings, particularly for extremely vulnerable populations.

## 1. Introduction

Handwashing is a key non-pharmacological intervention for stemming the spread of infectious diseases like COVID-19. Yet approximately 3 billion people, mostly in developing nations, lack access to basic handwashing facilities [1]. Brazil, one of the countries with the highest number of COVID-19 cases and deaths globally [2], is home to a large contingent of people who lack access to safe water [3]. Limited access to water, sanitation, and hygiene (WASH) has profound negative impacts on human health [4,5]. Aside from WASH insecurity, inadequate WASH can result in various morbidities, including diarrheal diseases and those related to water resource management, such as malaria and dengue [6]. Additionally, inadequate hand hygiene can facilitate the spread of infectious diseases, which has been exacerbated during the COVID-19 pandemic [7].

Brazil recently passed a bill to include water as a human right in the Constitution, but it has yet to be promulgated [8]. Currently, water is considered a ‘public good’ and its management is shared by federal, state, and municipal governments. Nonetheless, the fundamental rights to life, dignity, and health—all dependent on access to safe WASH—are recognized in Brazil’s Constitution and the federal units—the states—are mostly responsible for water supply within their territories [9]. Despite having the largest supply of freshwater in the world, universalization in WASH access in the country is far off-track [10]. As of 2021, 31 million Brazilians lacked access to safe water at home and about 93 million lacked basic sanitation [11].

Given that public entities have not been meeting their responsibilities, civil society organizations (CSOs) stepped in during the COVID-19 pandemic to ensure that vulnerable populations could practice hand hygiene and protect themselves and others from the coronavirus disease, putting the ‘public’ in ‘public health’ [12,13,14,15]. In the WASH sector, civil society is seen as crucial in addressing the needs of the most vulnerable in the provision of aid and equitable distribution of water services [10]. During the COVID-19 pandemic, even CSOs with differing mandates pivoted to developing strategies that responded to the public health crisis by capitalizing on their ability to orchestrate networks and resources inside and outside assisted communities [12].

Our research problem lies in understanding the role of CSOs in helping populations lacking access to safe WASH in the context of infectious disease outbreaks in low- and middle-income countries (LMICs). Despite the centrality of these actors in low-income settings, the literature on this topic is limited and those few studies that do exist focus on the HIV-WASH nexus, mostly in Sub-Saharan Africa [16,17]. Our paper contributes to filling this gap and expanding the focus to other geographical regions by using the COVID-19 pandemic and its effects in a water-rich nation like Brazil as a case study.

Aiming to understand the role of CSOs in supporting vulnerable populations during the COVID-19 pandemic, our research asks, firstly, how do leaders of civil society organizations perceive the impacts of the COVID-19 pandemic on populations with limited access to safe WASH in Brazil? Secondly, what actions have CSOs implemented to support vulnerable populations during the COVID-19 pandemic? Finally, what is the perceived level of success of these actions? To answer these questions, we conducted in-depth virtual interviews with CSO leaders working with populations with limited access to WASH in the metropolitan region of Rio de Janeiro.

This research is informed by the political ecology of health (PEH) framework, which aims to systematically understand social and environmental causes and consequences of disease, health, and wellbeing [18,19]. In this sense, our research understands health outcomes as products of political decision-making processes that influence the distribution of environmental and social determinants of health. This allows us to look at CSOs as political actors who determine the allocation of water and other health-promoting services. CSO leaders’ perceptions of the political economy of the studied region indicate a pathway to uncovering hidden political agendas that produce and reproduce health inequities. PEH also offers the concept of places of disease, which are areas of increased disease vulnerability and limited health decision-making options [19]. In this paper, we frame the marginalized communities under study as such places, produced by historical, social, and spatial processes. Moreover, this framework guides our exploration of different local understandings of COVID-19, discursively represented by different political actors such as public authorities and CSO representatives.

This paper is organized into five sections, including this Introduction. Section 2 describes the research design and methods, contextualizing health inequities in the study site. Section 3 presents results as they map onto our research questions. Section 4 contextualizes the results within the relevant literature while identifying potential limitations as well as contributions. The final section presents conclusions and reflections of lessons learned as well as transferability and future research directions.

## 2. Materials and Methods

**Figure 1:**
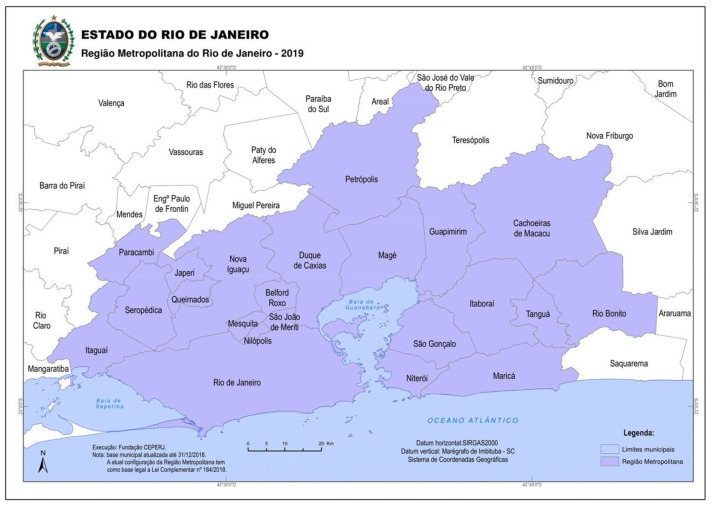
Map of the Metropolitan Region of Rio de Janeiro. Source: Fundação CEPERJ (2019). The figure is in the public domain, produced by the government of the state of Rio de Janeiro. Available at: http://arquivos.proderj.rj.gov.br/sefaz_ceperj_imagens/Arquivos_Ceperj/ceep/informacoes-do-territorio/cartografia-fluminense/Mapa%20da%20Regi%C3%A3o%20Metropolitana%20do%20Rio%20de%20Janeiro%20%E2%80%93%202019%20-%20CEPERJ.pdf, Accessed on 13 April 2023.

Rio de Janeiro is the capital city of a homonymous state. The city’s metropolitan region (MR) is comprised of 22 municipalities (Figure 1) home to 13.9 million inhabitants [20,21]. Baixada Fluminense is a sub-region within the MR comprised of the 11 municipalities located west of and including Duque de Caxias (see Figure 1). Discursively, Baixada Fluminense was constructed as the Periphery of the state capital, being known for high rates of violence and inequities in access to public services [22]. Although some parts of the region have seen an increase in economic opportunities, the peripheral populations are still largely dependent on the Capital [23]. Daily, approximately 2 million residents of the MR commute to Rio de Janeiro to work and access health, education, leisure, and other services [24].

Brazil’s National System of Sanitation reports that water supply in this region is above the national average (84%), reaching 91% of the population. However, this does not reflect water quality or the regularity of service. For example, 12 municipalities in the region are below the national average for water provision. Some, like Paracambi, Japeri and Seropédica are considerably below that number (71%, 68%, and 72%, respectively). Sanitation is even more limited; nine municipalities have no sewage treatment and only the Capital and three other municipalities treat more than 50% of their sewage [24].

When the COVID-19 pandemic started, the population of the Capital and Baixada Fluminense were already experiencing changes in the smell, taste, and color of their water. The state water company—CEDAE—communicated that these changes were caused by the presence of geosmin, an organic substance produced by cyanobacteria. The substance is non-toxic, but its presence is an indicator of a potential increase in levels of cyanotoxins, produced by the same microorganisms that generate geosmin [25]. This condition led the residents in this area to buy bottled water, incurring an increased economic burden.

This research was designed as a case study of the metropolitan region of Rio de Janeiro, selected because it represents a ‘perfect storm’ scenario—combining high rates of COVID-19 infections, a water crisis, insufficient state action to address the health crisis, and significant initiatives from CSOs to provide WASH access and other basic services. The unit of analysis—CSOs providing WASH access—is embedded within the research questions, which investigate community-based initiatives led by civil society organizations. Purposive sampling was implemented to allow for maximum variation and contextual differences in the level of success of the WASH interventions under scrutiny.

To address our research questions, we conducted virtual in-depth interviews (*n* = 15) with key informants (KI) in 15 different CSOs working with populations with limited access to WASH. The initial sample size was calculated to reflect two types of organizations (those that pre-dated the pandemic and those formed during the pandemic), CSOs operating in both the Capital as well as the Periphery, and those assisting either the homeless/inadequately housed, those of low income, or women. As the recruitment process unfolded, we began to recognize an overlap in service populations as well as the inclusion of other vulnerable populations. Interviewing continued until each of the selection criteria was satisfied, and saturation was reached, meaning that no new themes emerged from the analysis of the data. The resulting sample size is consistent with recommendations from recent literature [26].

After receiving ethical clearance (University of Waterloo Office of Research Ethics #42540), an inventory of CSOs with WASH-related activities in Rio de Janeiro (those donating water and hygiene kits, constructing water infrastructure, etc.) was developed; 37 prospective CSOs whose work was promoted in two main newspaper websites in the country [13,14] were contacted via e-mail or telephone based on contact information available on their websites and social media accounts. Participants were asked to refer the project to other CSO leaders whom they thought could contribute to the study.

During recruitment, prospective participants received an information letter that detailed the research objectives and design, as well as potential risks and benefits. Consent was obtained orally at the time of the interview, when participants had their rights reiterated prior to agreeing to initiate the interview and in writing via e-mail when they agreed to participate in the research. All interview transcripts were anonymized by the researcher before proceeding with data analysis. Participants were referred to as ‘KI’ and a number according to the order in which they were interviewed. Respective CSOs are referred to in the transcripts using the same number (e.g., KI1 and CSO1). Interviews were conducted (and recorded with permission) in Portuguese from January to April 2021 using Cisco Webex and were transcribed verbatim for subsequent thematic analysis. Conversational- style interviews included questions and probes related to five areas: background information about interviewees and their organization, WASH services in assisted communities, the impact of the COVID-19 pandemic, vulnerable populations, and barriers to action. A coding scheme was developed using both deductive (to address the research questions) and inductive (themes emerging from the transcripts) themes for subsequent line-by-line coding of the transcripts in NVIVO 13 [27]. Two members of the research team conducted inter-rater reliability coding of 10% of the transcripts, obtaining a level of agreement close to 90%.

Data were analyzed through pattern notation, making contrasts and comparisons, clustering, and counting [27]. Counts are reported in table form not to minimize the power of qualitative data, but to represent the relative importance of themes and sub-themes reported with respect to frequency of mention. Themes are punctuated with direct quotations from the CSO leadership.

## 3. Results

### 3.1. Effects of the COVID-19 Pandemic on WASH-Deprived Populations

Participants reported both direct and indirect effects of the COVID-19 pandemic on populations with limited access to WASH. Among direct effects (Table 1), participants reported both high and low rates of COVID-19 infections in the assisted populations. This happened partially because those who reported low morbidity were referring to the homeless populations. They believe that, when the lockdowns were implemented, this group was further isolated from those who pass by and indoor spaces. However, as these measures were lifted, KIs noted an increase in the number of homeless individuals contracting COVID-19:

*I have been hearing stories of the homeless population being more contaminated. This was when everybody started going to the beach, going out, gathering at parties*.

(KI1, serving the homeless in the Capital)

**Table 1:** Direct effects of the COVID-19 pandemic on assisted populations according to CSOs.

Sub-Theme	Participants Mentioning (*n* = 15)	%	Number of Mentions	%
High rate of infections observed	5	33%	7	30%
Low rate of infections observed	4	27%	6	26%
Unknown rate of infections	3	20%	3	13%
TOTAL			23	100%

Another explanation for this ambiguity is limited testing and sub-notification. Participants further expressed that testing was not easily accessible, and no governmental testing campaigns were targeting those of low income and/or those inadequately housed. For this reason, CSOs working with low-income families reported that the rates of infection are simply unknown:

*There is no data because the issue of sub-notification is grave. But we see through social media, there is not a day that you go online and there isn’t someone grieving. I think this is a great indicator*.

(KI11, serving those of low income in the Periphery)

Slum residents were more affected by the disease according to KIs. KI8 reports that in an irregular settlement with 40 families, 22 individuals reported COVID-19 symptoms, but none suspected they had the virus. The participant highlighted that the issue of disinformation aggravated the diffusion of the virus.

The indirect effects were more diverse and complex, reflecting the social, economic, and political factors that comprise the political ecology of COVID-19 (Table 2).

Income loss was the most frequently mentioned concern (Table 2); KIs report that many low-income individuals in Rio de Janeiro were not formally employed before the pandemic. With the lockdown, informal workers lost their livelihoods. Housekeepers, for example, were no longer working when their employers were respecting social isolation rules. Street vendors saw a decline in those who pass by, being deprived of selling goods in and near public transport stations. KI15 reports that single mothers were particularly affected:

*It was harder for women because some worked doing hair, nails, a lot of interpersonal contact. In the beginning, people didn’t want close contact* .

(KI15, serving those of low income and the homeless in the Periphery)

Additionally, companies were allowed to lay off or terminate employees’ contracts without proper remuneration, and CSOs had to suspend in-person income generation programs. As a result, KIs commonly reported that these vulnerable groups became food insecure:

*Hunger arrived before the virus in slums because the population that lived without formal employment earned at lunchtime what they would buy for dinner, and they lost their income*.

(KI3, serving the slums and those of low income in the Capital and Periphery)

“Generalized fear or confusion” was frequently mentioned by KIs across organizations. They reported assisted populations were initially afraid of getting sick, but also confused by the fact that the members of CSOs were wearing personal protective equipment (PPE) and that the streets were empty. This was the case among individuals in condition of homelessness:

*In the beginning, they were scared because we came with gloves, masks, face shields, etc. This caused a lot of surprises and distancing*.

(KI1, serving the homeless in the Capital)

Some KIs observed that certain groups gained income because of the financial emergency aid provided by the federal government. Deemed reasonable by some of the participants, the financial aid of BRL 600.00 (approximately USD 135.00)—reduced to BRL 300.00 after six instalments—helped support low-income households in 2020 [28,29]. During the period of data collection (January to April 2021), this financial aid was no longer provided, being later renewed for six months, with reduced instalments of BRL 150.00 [30]. The program’s temporary aspect and its progressive reduction in value compromised the income gain that was initially seen among the poorest:

*I heard many stories of people who left the streets and were able to pay the rent of a small room with the emergency aid. After its suspension, they could no longer pay rent*.

(KI1, serving the homeless in the Capital)

Eight participants reported that, during the pandemic, their organizations were sought by more people in need of assistance. Others noted that individuals with different backgrounds were joining their initiatives. KI10, whose organization normally operated at nighttime, noticed more women spending the nights on the streets. In her experience, before the pandemic, women used to be able to find shelter at night due to the increased risk of sexual violence:

*I observed a very large increase in women on the streets. Some live in nearby communities and end up commuting to get food for their families. And women with kids and without partners, which is a new profile*.

(KI10, serving the homeless in the Capital)

Other individuals who sought assistance were informal workers, especially street vendors. This group was also comprised of delivery persons working for food delivery apps, indicating the precariousness of informal employment and an immense irony: those who delivered food were starving. KIs remarked that, with the closure of businesses and people working from home, street vendors lost their income, and some started living on the streets:

*Everybody observed an increase in service. We had around 800 weekly actions. Suddenly it jumped to 1200. The street population remained the same, but we had an increase in informal workers*.

(KI10, serving the homeless in the Capital)

Finally, KIs working with slum dwellers reported intensified police intervention, already considered an issue before the pandemic. Some expressed that the state was present in slums only through the police. People in condition of homelessness were also repressed by the police, with a narrative to contain gatherings and to enforce the use of PPE. KI1 commented that these groups were being overlooked by distinct levels of government and not being provided with the means necessary to follow public health measures:

*There were violent actions in Rio de Janeiro where homeless people were fined for not wearing masks. Keep in mind that the mayor decided to buy paper masks that looked like birthday hats for the vulnerable population*.

(KI1, serving the homeless in the Capital)

Additionally, KIs suggested that lockdown measures particularly affected women and LGBTQIA+ (lesbian, gay, bisexual, transexual, queer, intersexual, asexual, aromantic, and related identities) individuals who suffer from domestic abuse in low-income settings because it confined them to being at home with their aggressors (commonly partners and parents, respectively). This perception is aligned with research that reveals that social isolation has contributed to increasing gender-based violence in domestic settings all throughout Latin America, in addition to highlighting aggravating factors such as elevated drug and alcohol consumption from male partners [31]. Similarly, while lived experiences of discrimination among LGBTQIA+ people are heightened compared to the general population, the lockdowns represented an additional source of stress for those who feel ostracized from their families due to their sexual or gender identities [32]. One of our KIs shared:

*Organizations that welcome LGBTQIA+ when they are kicked out of their houses had to be closed during lockdowns. So, LGBTQIA+ people who were already vulnerable because they had to live with violent parents, were now having to social isolate with them*.

(KI12, serving the homeless and those of low income in the Capital)

### 3.2. CSO Initiatives during the COVID-19 Pandemic

Regarding the effects of the pandemic on CSOs, most participants indicated their organizations had to either suspend, reduce, or adapt in-person activities (Table 3). Some organizations, like CSO2, who worked primarily with advocacy prior to the pandemic had to suspend their demonstrations in legislative and executive bodies at the municipal and state levels (the information under square brackets has been added by the authors for clarification):

*We adapted. We couldn’t be face to face with the people we were dealing with. Before, I would go to CEDAE [state water company], to the state assembly, watch the deliberations and confront the decision-makers*.

(KI2, serving the slums in the Capital)

**Table 3:** Effects of the COVID-19 pandemic on CSOs’ work.

Sub-Theme	Participants Mentioning (*n* = 15)	%	Number of Mentions	%
Suspended in-person activities	8	53%	17	20%
Reduced or adapted in-person activities	7	47%	12	14%
Increased scope or area of focus	8	53%	11	13%
Changed focus to relief aid	8	53%	10	12%
Reduced connection with assisted population	5	33%	9	11%
TOTAL			83	100%

Changes in CSO initiatives were linked to the effects of the pandemic on assisted populations. KI2 mentioned a program that aimed to offer counseling to women and LGBTQIA+ persons who were victims of domestic violence. With the pandemic, it had to be offered online or via telephone, which was perceived to hinder its efficacy:

*We thought long and hard about how are we going to propose therapy from home since it’s usually the space where people are abused. Are people going to feel free to speak inside the house?*.

(KI2 serving the slums in the Capital)

For the homeless population, the suspension of in-person activities meant that basic services, such as water and sanitation, were even harder to access. KI5 explained that her organization’s itinerant showers were shut down for nine months. Similarly, KI6 who works with low-income families had to suspend dental care and in-person classes, leaving children without access to education.

In this aforementioned scenario, CSOs adapted their activities to comply with public health measures and safeguard communities. Food and water donations were offered by CSO13 earlier in the morning to avoid large gatherings. They also started giving food packages that were prepared in advance at volunteers’ residences, so that recipients could disperse quickly. Most organizations implemented social distancing during their activities, distributing and demanding the use of masks and hand sanitizers. Other activities such as classes for children were offered by CSO6 in smaller groups in open spaces.

Many organizations that existed before the pandemic focused on a diverse set of activities (Table 4). However, during this health crisis, they pivoted toward relief aid through the donation of food, water, PPE, and hygiene materials (Table 5). Caring for basic needs became their primary focus, which was perceived as negative because other aspects of human development were left unattended:

*We were frustrated for not being able to perform our regular activities. Our organization’s mandate was never to provide emergency relief, to donate meals, etc*.

(KI10, serving the homeless in the Capital)

**Table 4:** CSO initiatives before the COVID-19 pandemic.

Sub-Theme	Participants Mentioning (*n* = 15)	%	Number of Mentions	%
Formal education	10	67%	15	18%
Art and culture	6	40%	12	14%
Health support	7	47%	12	14%
Income generation	7	47%	9	11%
Political incidence	4	27%	9	11%
Food donation	3	20%	7	8%
WASH	5	33%	7	8%
Community development	5	33%	7	8%
Sheltering or housing	3	20%	4	5%
Research	1	7%	1	1%
Facilitating access to assistance programs	1	7%	1	1%
TOTAL			84	100%

**Table 5:** CSO initiatives during the COVID-19 pandemic.

Sub-Theme	Participants Mentioning (*n* = 15)	%	Number of Mentions	%
Food donation	13	87%	33	11%
Partnering with local/regional CSOs	12	80%	28	10%
Donation of hygiene kits	13	87%	27	9%
Water donation	9	60%	22	8%
Raising awareness about COVID-19	9	60%	19	7%
Donation of PPE	11	73%	16	5%
Fundraising for relief aid	8	53%	15	5%
Raising awareness about hygiene practices/public health measures	5	33%	10	3%
Partnering with communities/individuals	8	53%	10	3%
Partnering with private organizations	6	40%	9	3%
Formal education	5	33%	9	3%
TOTAL			292	100%

CSOs also donated hygiene kits to help contain the spread of COVID-19 (Table 5). The kits consisted of soap, hand sanitizer, disinfectants, facial masks, and other regular hygiene products—toilet paper, menstrual pads, detergent, etc.

Although formal education was less prevalent during the pandemic, other educational initiatives took place. Nine CSOs tried to raise awareness about COVID-19 and five educated assisted populations about public health measures and hygiene practices (Table 5). KIs described distributing printed materials (e.g., banners, leaflets, booklets, stickers) that informed the assisted communities on how COVID-19 contamination could occur and how it could be prevented. These printed materials were supplied by the World Health Organization (WHO) and Instituto Oswaldo Cruz, a Brazilian institute for biological research and development. Prevention practices included adequate hand washing, masking, social distancing, avoiding sharing cigarettes, cups, and other personal objects, etc. Additionally, they delivered the same information orally, whenever donations or other interactions were taking place:

*Many groups made banners in the favelas stressing the importance of handwashing and prevention*.

(KI2, serving the slums in the Capital)

KI12 stressed the importance of oral communication when engaging further marginalized groups, such as LGBTQIA+ individuals in the condition of homelessness. She believes that, in their case, mistrust of governmental agencies acts as a barrier for following public health measures, and hearing from their peers at CSO12 helps increase compliance. KIs expressed that such activities were important to combat disinformation that was widely circulated in WhatsApp groups and by political leaders.

Almost all KIs mentioned working with partner organizations during the pandemic (Table 5) and many indicated not being able to work alone. Local grassroots organizations played a vital role in this scheme, since they better understand each community’s needs, territories, and norms. This is the case of CSO15, which expanded its area of focus from the Capital to the metropolitan region. KI15 expressed that she contacted local organizations in low-income communities because they knew the socioeconomic profile of the residents and could help allocate resources. Another arrangement was that of CSO10 who connected multiple organizations to offer different services simultaneously:

*We had partnerships with different organizations to provide showers or services of documentation. When the opportunity shows up, we provide the service*.

(KI10, serving the homeless in the Capital)

Other CSOs that participated in our study were conceived as the combination of several organizations, or as ‘umbrella’ agencies who supported other institutions. They utilized their networks to fundraise and transfer resources to local groups. This was the case of CSO3 and CSO2, who worked as hubs for donations for several other CSOs. When asked if she partnered with other CSOs, KI5 replied:

*I only work in partnership. I don’t see another way without partnerships. When someone leaves the streets definitively, other projects acted there so that they could be successful*.

(KI5, serving the homeless in the Capital)

While only five KIs mentioned working in WASH initiatives before the pandemic, nine became centrally involved in water donation activities due to inadequate access for these populations (Table 6). Prior to the onset of the pandemic, the homeless population accessed WASH services via local businesses, churches, and other organizations that were closed during the lockdown. Low-income families in the Periphery often faced intermittent or inadequate water supply, which became exacerbated by the lockdowns. In this context where frequent handwashing was virtually impossible, CSOs had to donate water or build WASH infrastructure where needed:

*We always had to supply the community with water. We started installing some very large water tanks with lids. And each family takes what they need. The water truck comes, fills it up, the water stays in a covered container, tidy, and clean*.

(KI6, serving the slums in the Periphery)

**Table 6:** Water supply among assisted populations.

Sub-Theme	Participants Mentioning (*n* = 15)	%	Number of Mentions	%
No direct supply	12	80%	22	61%
Intermittent supply	4	27%	12	33%
Regular supply	2	13%	2	6%
TOTAL			36	100%

The community supported by CSO6 is characterized by several WASH-related obstacles. Located in a former landfill, the soil is contaminated by slurry and the underground water is considered unfit for human consumption. Residents are underserved by the state company and not connected to water or sewage networks. According to KI6, they only have water from Sunday to Wednesday, and it only flows at night from one communal water collection point. Sewage treatment is non-existent, and most houses do not have toilets. Although not always combined, these obstacles are present in other areas, having been aggravated by the water supply crisis that affected the region. KI8 noted that other irregular settlements often have improvised water infrastructure, which affects the quality of the water accessed. Participants observed that even when families are housed and connected to the water network, the water supply is not reliable, and residents go weeks or months without water.

No key informants mentioned that the communities they served had ‘safe piped water’ (Table 7). Instead, participants reported that, before the water crisis, they had either unsafe piped water, unsafe water from alternative sources, or safe water from alternative sources. KI6 claimed that even where underground water is contaminated, some residents utilized well water or stored it in uncovered buckets or metal cans that facilitate contamination.

Few participants thought that the water quality worsened during the water crisis (Table 8). Those who did worked with populations in condition of homelessness. KI1 heard complaints about the taste and color of the water from these individuals, as well as cases of diarrhea. Conversely, those who did not observe the aggravation of water quality only thought so because they believed the water remained the same, i.e., unsafe. KIs expressed that because these communities do not receive piped water, they continued to access it from unsafe alternative sources. Those who are housed were indeed affected by the crisis, but KIs believed that the water quality was compromised even before the contamination issues were brought forward:

*There’s nothing else left to affect the street population. If the water is good, they don’t have water. If the water is bad, they don’t have water. Those who donate bring good water*.

(KI9, serving the homeless in the Capital)

**Table 8:** Water quality during the water crisis.

Sub-Theme	Participants Mentioning (*n* = 15)	%	Number of Mentions	%
Remained unsafe	5	33%	5	63%
Worsened	2	13%	3	38%
TOTAL			8	100%

Access to sanitation and hygiene facilities were even more limited (Table 9); the homeless lacked access to toilets, hygiene facilities, and showers:

*We don’t have public washrooms or sinks in the city. They end up bathing in the sea, in waterfalls or paying to shower somewhere else. Often, they must choose between the food or the shower*.

(KI10, serving the homeless in the Capital)

**Table 9:** Sanitation and hygiene services in assisted communities according to CSOs.

Sub-Theme	Participants Mentioning (*n* = 15)	%	Number of Mentions	%
No washrooms	10	67%	20	44%
No sewage treatment	5	33%	8	18%
Hygiene facility provided by others	3	20%	6	13%
Improvised washrooms	2	13%	3	7%
Shared washrooms	2	13%	3	7%
Paid hygiene facilities	2	13%	3	7%
Inadequate sewage treatment	2	13%	2	4%
TOTAL			45	100%

Similarly, some low-income households in the Periphery have only improvised washrooms and no sinks. It is also rare for families to have piped sewage; this lack of sanitation infrastructure poses risks to health and the already limited water network.

### 3.3. Perceived Level of Success of Interventions

KIs reported the perceived success of their COVID-19 interventions (Table 10).

One initiative perceived as successful was the installation of mobile sinks. This was spearheaded by CSO5 and, according to them, 134 sinks were installed in the Capital and metropolitan region:

*It consisted of sinks made of plywood and plastic, with a tap and all, where the homeless population would wash their hands. It was brilliant*.

(KI13, serving the homeless in the Capital)

These sinks also helped building a sense of community among the users and the public who supplied water, soap, and other materials, contributing to ‘general public engagement’ (Table 10). However, maintaining the sinks in some locations was difficult:

*In deserted places, the sinks were destroyed. Otherwise, the reception was good. They truly embraced it*.

(KI5, serving the homeless in the Capital and Periphery)

Some KIs also indicated that the number of donors and the value of donations increased during the pandemic (Table 10), which they explained as civil society being sensitized by exacerbated social inequities. KI2 noted that their campaigns raised more than BRL 600,000 (~USD 135,000), while CSO3 was one of the biggest donors in the country, having raised more than BRL 6,000,000 (~USD 1,350,000) for the creation of new intensive care unit (ICU) beds:

*In many campaigns, we knew how to capture this feeling, this driving force to help and we redirected it to donations*.

(KI2, serving the slums in the Capital)

Compliance with public health measures was seen both as successful and unsuccessful. KI8, for example, explained that, initially, people would not wear masks in open spaces or socially isolate when sick. She noted, however, that after encountering CSO8, people were aware of such measures and sought the organization for health information. Nonetheless, adherence to public health measures was mostly considered unsuccessful:

*They don’t wear masks. There is no form of isolation. In Jardim Gramacho, you go to a drug store and not even the receptionists wear masks*.

(KI6, serving the slums in the Periphery)

A frequent barrier to compliance with these measures was disinformation. KIs were frustrated with political leaders at all levels who spread false information, minimizing the severity of COVID-19 and the importance of non-pharmacological interventions. So, although CSOs were intensively working to contain the spread of the coronavirus, major political actors worked as counterproductive forces:

*At the beginning of the pandemic, the president spread that it was just a flu, that we would only lose 800 people. That heavily affected the way people saw the pandemic*.

(KI15, serving the homeless and those of low income in the Periphery)

KIs also commented on governmental support to vulnerable populations. Amid some relief aid initiatives, the third most frequent response was ‘non-existent’. Moreover, most government actions were seen as bad (81%) while only five of 36 mentions were seen as acceptable and two as good.

Government initiatives classified as ‘good’ included food donations and the creation of a shelter for LGBTQIA+ persons in the condition of homelessness. Deemed as acceptable was the financial emergency aid. All the other actions, classified as bad by KIs, were considered too sporadic or inadequate to address the real needs of vulnerable populations. In this scenario of inadequate or inexistent support from public authorities, paired with disinformation from political leaders, KIs felt that the civil society had to take responsibility for the provision of basic public services, formally a role of the state:

*The government took a long time to reach these people, so the NGOs and collective groups provided aid to them*.

(KI11, serving those of low income in the Periphery)

## 4. Discussion

The scenario described in the last paragraph was categorized by Ortega and Orsini (2020) as “governance without (central) government” since Brazil’s president at the time—Jair Bolsonaro—had actively undermined the severity of the COVID-19 pandemic and questioned public health measures to contain it [33,34]. This governance was played out by multiple actors, including some local governments, civil society organizations, self-organized community groups in favelas and Indigenous communities, as well as the traffic and militias [33]. These authors noted that Bolsonaro and the members of his cabinet intensified their abdication of responsibility for public health governance during the pandemic, mainly through denialism of scientific evidence, dissemination of fake news, and freezing of public health funding [33]. Functions ascribed to the public mechanism were then transferred to actors outside of the state, most commonly to civil society organizations [33,35]. Our case study found very similar results as we will discuss in this section.

This research explored the effects of the COVID-19 pandemic in populations with limited access to WASH in Rio de Janeiro using CSO leaders as key informants. We found, firstly, that vulnerable populations were directly affected by the virus, causing generalized fear and high rates of infection. While this was true for most low-income communities, some key informants observed few infections among individuals in condition of homelessness. A possible explanation was the lack of data due to limited testing. This is consistent with Fouad and colleagues’ findings in Lebanon, where there was no systematic testing of Syrian refugees in irregular settlements [36]. Similarly, a nationwide study that investigated social vulnerability in Brazil during the COVID-19 pandemic listed limited testing as one of the challenges to determine rates of infection and mortality [37]. On the other hand, another study described how slum dwellers in Dharavi, India, avoided the first wave of the pandemic with a strategy that relied heavily on testing and contact tracing [38].

Secondly, our findings indicate that the pandemic exacerbated pre-existing social inequities in Rio de Janeiro, undermining vulnerable populations’ capacity to protect their health. Consonant to that is research that shows slum residents as the first individuals to lose their livelihood when lockdowns were implemented in India and Ghana [39,40,41]. De Paula and colleagues’ interviews with homeless persons in Rio de Janeiro confirm our findings that social isolation created new challenges for this group given that the absence of those who pass by, the closure of local businesses, and the suspension of humanitarian actions resulted in reduced donations and casual economic opportunities [12]. The Brazilian Institute of Geography and Statistics reported that unemployment in Brazil rose from 10.5% to 14.4% between May and September 2020 [42]. However, there is evidence that this rate was higher among poorer communities. A study with 457 heads of families in Brazilian slums reported an unemployment rate of 45% within the same period. Additionally, 46.5% of the families faced challenges to buy food [43].

As the lines for receiving food donations grew, so did disease vulnerability. Low-income populations in developing countries faced several challenges to comply with public health measures implemented during the pandemic [12,44,45]. That includes the absence or inadequacy of hygiene facilities, the intermittency of water supply, and limited (or inexistent) space to socially isolate. A study conducted in 600 Bangladeshi slums showed a direct correlation between overcrowded households and shared WASH facilities, which puts slum dwellers at greater risk of contracting COVID-19 [46]. In Rio de Janeiro, these conditions were further complicated by a water crisis. This is a perfect storm of social and spatial processes shaping what Brian King called “places of disease” [19]. In this sense, the homeless populations and slum residents in Rio de Janeiro inhabit a place where disease vulnerability is exacerbated and health decision-making is compromised by the lack of basic services, which is an outcome of macro-economic, political, and environmental processes. Wasdani and Prasad argued that precarious conditions, combined with the migratory nature of informal employment, pose health risks to society at large [40]. Thus, addressing health inequities in the context of a pandemic advances population health in general.

In this study, the places of disease are not necessarily fixed in space. While slums and low-income communities are confined to a specific geographical area, homeless populations occupy the spaces that are available in the city. In their case, the place of disease is embodied. Their living conditions concentrate the outcomes of social and spatial processes that produce disease vulnerability, despite their location. For that reason, environmental determinants of health are not available because these populations are in a particular area, but because they are houseless: as stated above, “If the water is good, they don’t have water. If the water is bad, they don’t have water” (KI9, serving the homeless in the Capital).

In this scenario, CSOs met these groups’ pressing needs, changing their scope of action from multiple areas of development to relief aid. By having CSOs only focus on basic needs, vulnerable populations were left unattended in other areas because of the absence and inadequacy of governmental response. This is seen in other contexts of limited political engagement. In Medellín, Colombia, approximately 120 civil society organizations developed an emergency plan to minimize the impacts of COVID-19 in vulnerable communities [47]. In India, CSOs donated food to migrant workers and slum dwellers, and, in Rwanda, they installed handwashing facilities at bus stations [39,40]. In Zimbabwe, CSOs have worked alongside the national government and UNICEF to identify gaps in WASH services in healthcare facilities during the COVID-19 pandemic in an example of how governments can leverage from supporting CSOs and exploring their knowledge to inform public health policy [48].

Nonetheless, as exemplified by President Jair Bolsonaro’s claims that COVID-19 was a little flu [49], national and local decision-makers acted as obstacles to CSOs’ actions in Brazil. CSOs had to combat fake news being spread by powerful leaders aside from providing basic services. The political ecology of health seeks to understand how institutions shape the notions of diseases through discourse, and how these narratives are processed by local understandings [19]. In our case study, there were two competing discourses: one that represented COVID-19 as a serious infectious disease that should be prevented with non-pharmacological interventions, and one that diminished its seriousness and belittled said interventions [34]. The latter was defended by politicians in formal positions of power and the former by CSO representatives. A similar case was seen in South Africa during the HIV/AIDS epidemic when former South African president, Thabo Mbeki, imposed challenges to the broad distribution of anti-retroviral therapy to treat HIV. The political group in power contradicted science by questioning the link between HIV and AIDS. As seen in Rio de Janeiro, it was the civil society that stepped in, pressuring the government to make the treatment available nationally [19].

Misleading health information poses a significant challenge to public health, which became more evident during the COVID-19 pandemic (see, for example, [50]), with infamous declarations such as Donald Trump’s suggestion that the ingestion of bleach could be a cure for COVID-19 [51]. For that reason, the fact that CSOs sought to obtain the most up-to-date evidence-informed health information from reputable organizations such as the WHO can be seen as an important practice to be followed in other politically contested health crisis scenarios.

In this sense, CSOs not only had to take on the role of the state [33], but they also had to fight the government’s ongoing campaign to discredit up-to-date scientific information about COVID-19 and its prevention. Consequentially, these populations were left unattended in the areas that were previously tackled by civil society initiatives, revealing how this ‘governance without government’ logic was important—at least in the metropolitan region of Rio de Janeiro—even before the current health crisis.

There were clear gender-specific issues in our study. Research indicated that women, traditionally responsible for fetching water, were more exposed to COVID-19 as they had to gather around common water sources [37,52]. However, women were also at the forefront of the efforts to tackle the effects of the pandemic in Rio de Janeiro. Approximately three-quarters of the CSO leaders we interviewed were women (11 of 15). Nunes (2021) explains that multiple community-based organizations were created in Rio de Janeiro during the 1990s to improve the living conditions in favelas and the social recognition of women [53]. Thus, during the pandemic, women ahead of these organizations had strong networks outside and inside favelas that they could mobilize to provide emergency aid [53].

Our research showed the importance of pairing emergency aid initiatives with sensitization about health decision-making. By understanding the needs and rules of the communities they attend, CSOs can effectively convey health information to vulnerable populations. This is particularly important when disinformation is widespread by powerful political actors. Hence, CSO leaders and others involved in sharing health information need to occupy the virtual spaces where fake news is shared, such as WhatsApp. This communication might be more effective if conducted by people who are part of these groups and who share local understandings of health.

In scenarios of infectious disease outbreaks like COVID-19, public health measures must take into consideration the capabilities of vulnerable populations. The imposition of lockdowns had harmful effects on homeless populations and informal workers [40]. Even when social distancing is recommended, CSOs need to be supported so that they can continue their work with these groups safely. Recent research shows that zoonoses like COVID-19 are expected to become more common as hazardous interactions between humans, livestock, and wildlife intensify [54,55]. Therefore, societies need to be ready for future pandemics. Building reliable WASH infrastructure is part of that and, during the current health crisis, it was successfully achieved by CSOs in Rio de Janeiro. Thus, wherever housing demands cannot be met in the short term, governments must provide hygiene and sanitary facilities, as well as reliable water supply in easily accessible public spaces.

## 5. Conclusions

The COVID-19 pandemic has shone a spotlight on pre-existing global health inequities. Our study focused on the role of civil society organizations in responding to these inequities. Through the political ecology of health lens, we identified the vital role that these organizations had and illustrated the importance of considering multiple political actors in the study of the geographies of human health. Where formal institutions seem to act against the improvement of population health, bottom-up approaches indicate an alternative and powerful counterforce.

COVID-19 exacerbated pre-existing social inequities among vulnerable populations, undermining their ability to protect their health and the health of others. CSOs provided emergency relief aid but faced the counterproductive actions of public authorities who promoted a narrative diminishing the risks of COVID-19 and the importance of non-pharmacological interventions. CSOs fought this narrative by promoting sensitization among vulnerable populations and partnering with other stakeholders in networks of solidarity, playing a vital role in the distribution of health-promoting services. CSO initiatives were mostly seen as successful by CSO leaders except for the ones that aimed to raise awareness about health safety practices and the suspension of in-person activities.

A potential limitation of the research is the lack of investigation on the motivation behind the perceived inaction of different levels of government in Brazil. Moving forward, research in this field would benefit from combined top-down and bottom-up approaches that take into consideration various social forces in the construction of health and disease local understandings, and the distribution of health-promoting resources.

Nonetheless, CSOs are limited in providing support to vulnerable populations, especially during a pandemic. As discussed earlier, infectious disease outbreaks are expected to be more frequent in the future. It is the public sector’s responsibility to provide vulnerable populations with the tools necessary to protect their health and, therefore, population health at large. That entails addressing inequities in access to WASH, by guaranteeing universal access to safe water, as well as preventing water contamination through adequate sewage treatment. Moreover, it includes promoting equitable access to healthcare and health knowledge. Governments can benefit from partnering with CSOs to ensure that communities are autonomous in making healthy decisions and minimizing the deleterious effects of future pandemics on global populations.

## Figures and Tables

**Table 2:** Indirect effects of the COVID-19 pandemic on assisted populations according to CSOs.

Sub-Theme	Participants Mentioning (*n* = 15)	%	Number of Mentions	%
Income loss	9	60%	15	29%
More people seeking assistance	8	53%	9	18%
Generalized fear or confusion	7	47%	7	30%
Diversified profile of assistance seekers	4	27%	7	14%
Increased policing	3	20%	6	12%
Increased domestic violence	5	33%	6	12%
TOTAL			51	100%

**Table 7:** Water quality before the water crisis.

Sub-Theme	Participants Mentioning (*n* = 15)	%	Number of Mentions	%
Unsafe piped water	6	40%	7	47%
Unsafe water from alternative sources	6	40%	7	47%
Safe water from alternative sources	1	7%	1	7%
TOTAL			15	100%

**Table 10:** Initiatives perceived as successful vs. unsuccessful.

Sub-Theme	Participants Mentioning (*n* = 15)	%	Number of Mentions	%
Successful initiatives				
Installation of mobile sinks	4	27%	9	41%
Fundraising	3	20%	5	23%
General public engagement	3	20%	3	14%
Adoption of hygiene practices	2	13%	3	14%
Public health compliance	1	7%	1	5%
TOTAL			22	100%
Unsuccessful initiatives				
Public health compliance (other than masks)	5	33%	6	25%
Adoption of masks	4	27%	6	25%
Mobile sink maintenance	4	27%	5	21%
Suspended in-person activities	2	13%	3	13%
Solely providing relief aid	3	20%	3	13%
TOTAL			24	100%

## Data Availability

The data presented in this article are not publicly available due to privacy restrictions.
